# Effects of fluorescent tags and activity status on the membrane localization of ROP GTPases

**DOI:** 10.1080/15592324.2024.2306790

**Published:** 2024-01-25

**Authors:** Jingtong Ruan, Zihan Yin, Peishan Yi

**Affiliations:** Key Laboratory of Bio-Resource and Eco-Environment of Ministry of Education, College of Life Sciences, Sichuan University, Chengdu, Sichuan, P. R. China

**Keywords:** Cell polarity, ROP GTPase, moss, fluorescent protein tagging, membrane trafficking

## Abstract

Plant-specific Rho-type GTPases (ROPs) are master regulators of cell polarity and development. Over the past 30 years, their localization and dynamics have been largely examined with fluorescent proteins fused at the amino terminus without investigating their impact on protein function. The moss *Physcomitrium patens* genome encodes four *rop* genes. In this study, we introduce a fluorescent tag at the endogenous amino terminus of ROP4 in wild-type and *rop1,2,3* triple mutant via homologous recombination and demonstrate that the fluorescent tag severely impairs ROP4 function and inhibits its localization on the plasma membrane. This phenotype is exacerbated in mutants lacking ROP-related GTPase-activating proteins. By comparing the localization of nonfunctional and functional ROP4 fusion reporters, we provide insight into the mechanism that governs the membrane association of ROPs.

## Introduction

Cell polarity is essential for pattern formation and particularly important during plant development.^1^ One of the best-known polarity proteins in plants is Rho-of-plants (ROP) GTPase.^2–4^ ROPs belong to the Cdc42/Rho/Rac superfamily and fall into two subtypes, of which the type-I ROPs are present in all land plants and are anchored to the membrane via a canonical prenylation signal at the carboxyl terminus (C-terminus).^5^ Being asymmetrically distributed on the membrane, ROPs function to regulate many developmental processes such as the growth of pollen tubes and root hairs,^6,7^ the formation of the zig-zagged shape of pavement cells,^8^ and secondary cell wall patterning.^9^ The moss *Physcomitrium patens* and liverwort *Marchantia polymorpha* have recently emerged as new models for studying ROP signaling in basal land plants.^10^ Knockout *rop* mutants not only exhibit growth defects in tip-growing cells such as protonema cells and rhizoids but also show dramatic disorganization in cell division and tissue patterning,^11–14^ suggesting that ROP-mediated polarization is highly conserved in plants.

One key aspect of understanding ROP signaling is to know where ROPs localize and how their polar localization is achieved. Early studies employ immunostaining to examine ROP localization.^15,16^ With the advancement in live-cell imaging, ROPs are now tagged with fluorescent proteins to examine their dynamic recruitment and have revealed the formation of membrane nanoclusters that are critical for polarity establishment.^17–20^ As the prenylation signal is important for ROP localization^21,22^ and the tagging of ROPs with a C-terminal fluorescent protein severely impairs membrane association,^14^ most studies use an amino-terminal (N-terminal) tag to visualize ROP dynamics. However, a recent report^23^ and our independent study in the moss *P. patens* find that N-terminal tagging impairs the function of ROPs and reduces their membrane association. This highlights the need for caution when interpreting localization and dynamics data obtained with an N-terminal fluorescent tag. In addition, we show that, when ROP activity is upregulated, N-terminally tagged ROPs are relocated in cytosolic puncta-like structures that do not colocalize with vesicles. These findings suggest that cytosolic shuttling plays a critical role in regulating ROP localization.

## Materials and methods

### Moss strains and culture conditions

Moss plants used in this study were derivatives of the Gransden ecotype of *Physcomitrium patens* (previously known as *Physcomitrella patens*) and were cultured on standard BCDAT solid medium at 23 ~ 25°C under continuous white light. The propagation of plants was performed by inoculating tissue fragments either from protonemata or gametophores directly on BCDAT plates or distributing fragmented tissues on cellophane-laid BCDAT plates. The *rop1,2,3* triple mutant, *ropgap, ren* septuple mutant, and knock-in lines of N-terminally or internally tagged ROP4 with mNeonGreen protein (mNG) were generated previously.^14,24^

### Molecular biology and transgenesis

To generate knock-in lines of N-terminally tagged ROP4 with mNG in the *rop1,2,3* triple mutant or *ropgap, ren* septuple mutant, the same construct used by^14^ was transformed into the mutants. Correct insertion of the mNG coding sequence immediately downstream of the start codon via homologous recombination was confirmed by PCR amplification and DNA sequencing. To rescue the localization defects of N-mNG-PpROP4 in the *ropgap, ren* mutant, the plasmid pPY140 that expresses Cerulean-tagged REN fusion protein under the control of EF1α promoter was transformed into the *ropgap, ren* + N-mNG-PpROP4 mutant. The transformation experiments were performed as described previously.^14,24^ Briefly, ~30 µg of plasmids were linearized via restriction enzyme digestion, purified by ethanol precipitation, and transformed into protoplasts through the PEG-mediated transformation protocol. Stable transgenic lines were obtained by resistance selection and used for subsequent analyses.

### Live-cell imaging and image analysis

Imaging samples were prepared by inoculating a small piece of moss protonema tissues on a thin layer of solid BCD medium in 35-mm bottom-glass dishes. After 5 ~ 7 days of culture, sample dishes were directly placed on an inverted Axio Observer Z1 spinning-disk confocal microscope (Zeiss) to perform imaging experiments. Images were taken using a 10 × 0.45-NA, 40 × 1.30-NA, or 63 × 1.40 NA objective lens and a Hamamatsu camera controlled by the Zeiss Zen software (version 2.3, Blue Edition). The excitation/emission wavelengths were 488/517 nm (green) and 561/603 nm (red). Image processing and analyses were performed using the Fiji software (version 2.14.0). As cells of *rop1,2,3* + N-mNG-PpROP4 knock-in lines were super round, it is difficult to determine their growth direction. The longest axis was intuitively defined as the growth axis and used as a reference to measure cell length and width in this mutant. To quantify the membrane and cytosol intensity ratio, a single line with spline fit covering a ~ 6-µm region near the cell apex, where ROPs were mostly enriched, was drawn. Mean intensity was measured and compared with the mean intensity of a nearby region in the cytoplasm of the same size. To show fluorescence distribution along the apical membrane, an intensity profile was generated from a line region at one side of the membrane for each cell from the cell apex to the subapical region. Intensity profiles from different cells were averaged for quantification. Colocalization analysis was performed in Fiji using the ComDet plugin (version 0.5.5). The approximate particle size was set to 3 ~ 4 pixels and the intensity threshold was manually inspected to ensure most visible particles were detected.

## Structure prediction

For monomeric and dimeric structure prediction, full-length amino acid sequences of PpROP4 (Pp3c10_4950V3.1.p), PpRopGAP1 (Pp3c4_16800V3.1.p), RopGEF4 (Pp3c2_28420V3.1.p) and PpRopGDI1 (Pp3c3_32980V3.1.p) were used. For tetramer prediction, only the CRIB domain and GAP domain of PpRopGAP1 and the PRONE domain of PpRopGEF4 were used because full-length sequences often resulted in disorganized structures likely due to reduced prediction accuracy. The prediction was performed using Alphafold^25^ for monomeric proteins and AlphaFold-multimer for protein complexes.^26^ The source codes were available at https://github.com/google-deepmind/alphafold. Both relaxed and unrelaxed structures from five different models were obtained for each prediction and one of the most common structures or most compact structures was chosen for analysis and comparison. The experimentally obtained structure of Arabidopsis AtROP4 and AtRopGEF8 PRONE domain (PDB:2NTY) and Human HsRhoA (PDB: 5C4M) were used as a reference to assess the prediction accuracy of PpROP4. Structures were visualized in the Chimera software (University of California, San Francisco).

### Chemical treatment

To stain vesicles, 1 µl of FM4–64 stock (10 mM dissolved in DMSO) was diluted in 1 ml of sterilized water. 200 µl of the diluted FM4–64 (10 µM) was directly added to moss plants in 35-mm imaging dishes. The dishes were kept in the dark for 30 min. Subsequently, the FM4–64 solution was removed before imaging.

### Statistical analysis

Statistical analyses were performed using two-tailed unpaired student’s t-tests. The sample size (*n*) in each experiment is indicated in the main text or figure legends. A significant difference was determined when the p-value was less than 0.05.

## Results

**Figure 1.**
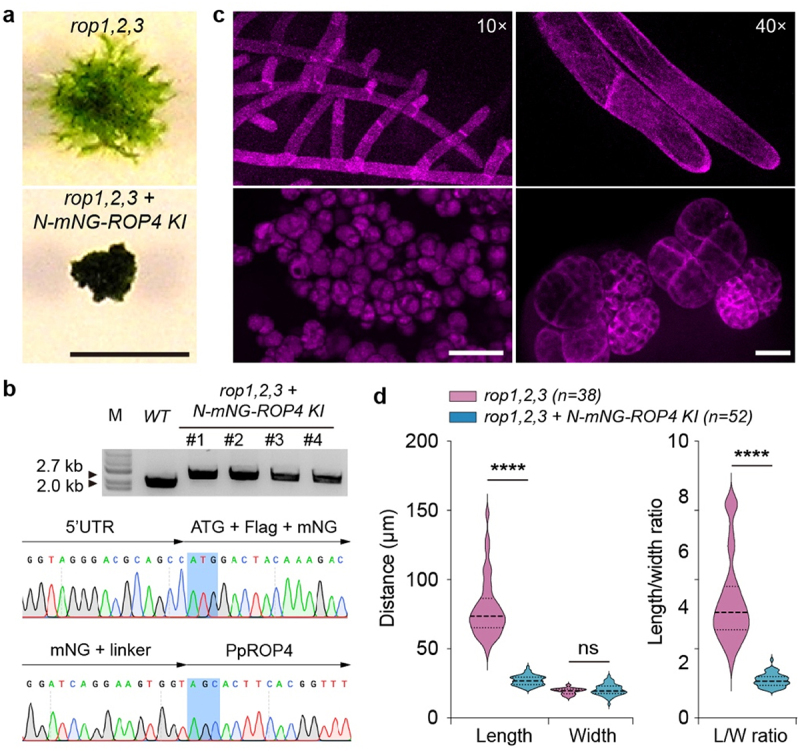
N-terminal tagging with a fluorescent protein tag strongly impairs ROP4 function in the *rop1,2,3* triple mutant background. **a** Representative moss colonies of *rop1,2,3* triple mutant and ROP4 knock-in (KI) lines. The ROP4 knock-in lines were generated by inserting the coding sequence of green fluorescent protein mNeongreen (mNG) immediately downstream of the ROP4 start codon via homologous recombination. Four independent lines were obtained and exhibited similar growth defects. Scale bar: 5 mm. **b** verification of KI lines via PCR amplification and sequencing. DNA sequencing confirmed the correct integration of mNG without introducing additional mutations. **c** morphology of protonema cells of *rop1,2,3* triple mutant (top) and ROP4 KI lines (bottom) under a 10× lens (left) or 40× lens (right) labeled by Lifeact-mCherry reporters. Cells in the KI lines were imaged on a glass slide, forming dispersed clusters under the pressure of the coverslip. Scale bars: left, 100 µm; right, 20 µm. **d** quantification of cell length, cell width, and length/width ratio. Data are presented as violin plots, showing the quartiles (thin lines) and the median (central line). Statistical analyses were performed using two-tailed student’s t-tests. ns, not significant. ****, *p* < 0.0001.

Compared with Arabidopsis, which has 11 *rop* genes, *P. patens* has only four *rop* genes.^27^ The four *rop* genes encode highly similar polypeptides and are functionally redundant.^11,14^ Previously, we generated higher-order *rop* mutants using CRISPR/Cas9-assisted genome editing technology and found that the *rop1,2,3* triple mutants exhibited mild growth and developmental defects compared with wild-type plants.^14^ When the coding sequence of the mNeonGreen fluorescent protein (mNG) was introduced to the N-terminus of endogenous ROP4 (N-mNG-PpROP4) via homologous recombination in the *rop1,2,3* triple mutant, the knock-in lines exhibited strong growth defects and could not develop into filamentous protonemata (Figure 1a-c), although N-mNG-ROP4 in the wild-type background did not influence plant development due to gene redundancy.^11,14^ Rather, all cells adopted a highly rounded shape and aggregated into irregular clusters (Figure 1c). These cells exhibited reduced adhesion to one another, and their cell walls were notably stiffer compared to *rop1,2,3* mutant cells. Furthermore, the mutants exhibited a marked inability to differentiate into gametophores (Figure 1a). We quantified the length and width of cells and found that cell length but not width was significantly reduced (Figure 1d), suggesting that cell shape phenotypes were largely caused by the strong loss of tip growth. These findings were consistent with the reported phenotypes of *rop1,2,3,4* quadruple mutants.^14,23^ Therefore, we concluded that N-terminal tagging of ROP4 with a fluorescent protein strongly impairs its functions in vivo.

**Figure 2.**
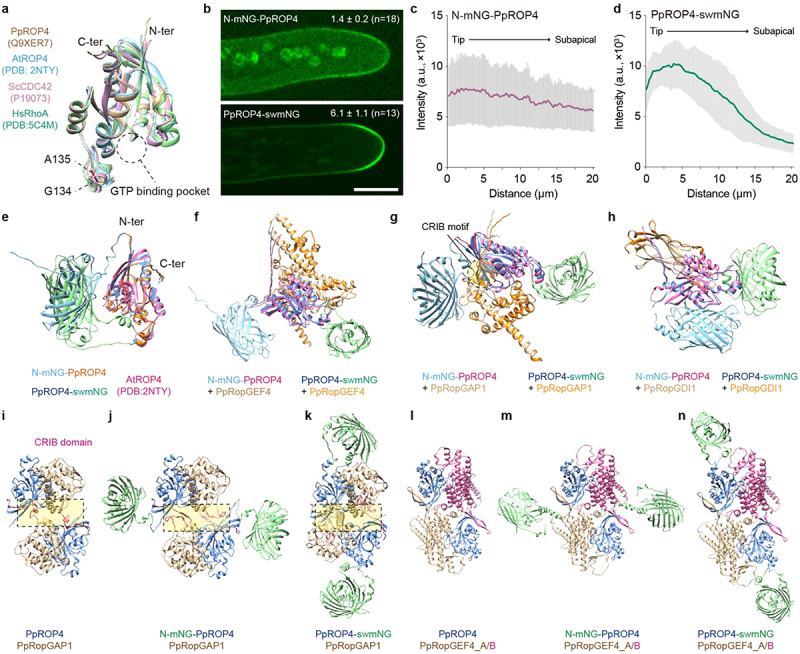
The localization of ROP4 at the plasma membrane is altered by an N-terminal mNG tag (N-mNG). **a** overlay of predicted structures of moss PpROP4 and yeast ScCDC42 and the experimentally obtained structures of Arabidopsis AtROP4 and human HsRhoA. All proteins exhibit a similar conformation. The N-terminal (N-ter) and C-terminal (C-ter) regions are indicated. The loop region of PpROP4 containing glycine 134 (G134) and alanine 135 (A135) is highlighted. The fusion of an mNG tag between G134 and A135 (swmNG) does not impair ROP4 function.^23^ **b** the localization N-mNG-PpROP4 and PpROP4-swmNG. The average membrane/cytosol intensity ratio for each genome type is shown. N-mNG-PpROP4, *n* = 18 cells; PpROP4-swmNG, *n* = 13 cells, mean ± SD. **c** intensity profile of N-mNG-PpROP4 from protonema cell tip to the subapical region along the plasma membrane. Intensity values were measured from 10 cells and shown as mean ± SD. **d** intensity plot of PpROP4-swmNG from protonema cell tip to the subapical region. Intensity values were measured from 11 cells and shown as mean ± SD. Note that there is a sharp decrease of signals around 15 µm distant from the tip. **e** the predicted structures of N-mNG-PpROP4 and PpROP4-swmNG. **f-h** the predicted dimeric structures of N-mNG-PpROP4 and PpROP4-swmNG with PpRopGEF4, PpRopGAP1, and PpRopGDI1. **i-k** the predicted tetrameric structures of PpRopGAP1 with PpROP4, N-mNG-PpROP4, and PpROP4-swmNG. The CRIB domain of PpRopGAP1 is shown in magenta. The dimeric interaction interfaces are highlighted in dashed boxes. **l-n** the predicted tetrameric structures of PpRopGEF4 with PpROP4, N-mNG-PpROP4, and PpROP4-swmNG. The two PpRopGEF4 subunits (A/B) are differently colored to facilitate visualization.

In both yeasts^28^ and *P. patens*,^23^ the fusion of Cdc42 or ROP4 with an internal fluorescent protein does not affect their function. Noticeably, PpROP4 fused with a sandwiched mNG (swmNG) in the loop region between Glycine 134 and Alanine 135 does not affect plant growth in either wild-type or *rop1,2,3* triple mutants.^23^ Accordingly, we generated PpROP4-swmNG (Figure 2a) and compared its localization pattern with N-mNG-PpROP4 in the wild-type background. Although both fusion proteins exhibited enrichment on the apical membrane in tip-growing protonema cells, the membrane signals of PpROP4-swmNG are much stronger (Figure 2b). Quantitative analyses revealed a gradual decrease of intensity from the apex to the subapical membrane of N-mNG-PpROP4 (Figure 2c). By contrast, there was a sharp decrease in signals of PpROP4-swmNG ranging from 10 µm to 20 µm distant from the apex on the membrane (Figure 2d). In addition, the intensity ratio between membrane and cytosol at the apical region of N-mNG-PpROP4 was significantly lower than that of PpROP4-swmNG (see Materials and Methods, Figure 2b). These results suggest that N-terminal tagging impairs the membrane localization and enrichment of ROP4.

We next asked how fluorescent protein tagging may impact the function and localization of ROPs. To this end, we predicted the structures of N-mNG-PpROP4 and PpROP4-swmNG using AlphaFold2.^25^ In both structures, PpROP4 exhibited a highly similar conformation to the experimentally obtained structure of Arabidopsis AtROP4 (PDB: 2NTY),^29^ and N-mNG and swmNG were both distant from the C-terminal prenylation site (Figure 2e). Therefore, the reduced membrane association may not be caused by defects in membrane anchoring. Similar to yeast Cdc42, the polar localization of ROPs requires activity control and involves ROP-related Guanine Nucleotide Exchange Factors (RopGEFs), GTPase Activating Proteins (RopGAPs), and Guanine Nucleotide Dissociation Inhibitors (RopGDIs).^4,5^ The current model suggests that ROPs must be dynamically activated and inactivated, and active ROPs should be less mobile than inactive ROPs to ensure cluster formation.^4,20,30^ To test whether protein tagging interferes with the binding of ROPs with these regulators in space, we obtained predicted structures of N-mNG-PpROP4 and PpROP4-swmNG in complex with PpRopGEF4, PpRopGAP1, and PpRopGDI1, respectively, using the AlphaFold2 multimer algorithm.^26^ As shown in Figure 2f-h, N-mNG and swmNG were placed at distinct positions in these structures, however, they did not tend to impact subunit binding in each heterodimer.

Because ROPs, RopGAPs, and RopGEFs could form clusters and membrane domains in vivo,^17–20,24,31^ and AtRopGEF8 and AtRopGAP2 have been reported to exist in tetramers with ROPs,^29,32,33^ we speculate that fluorescent tags may affect the formation of high-order complexes in space. Therefore, we predicted tetramer structures for PpROP4-PpRopGAP1 and PpROP4-PpRopGEF4, respectively, in the absence of an mNG tag, with N-mNG fusion, or with swmNG fusion for PpROP4. Plant RopGAPs contain a unique CRIB domain before the GAP domain.^5,34^ They could bind ROPs separately in vitro^24,34,35^ and possibly target one ROP molecule simultaneously.^32,33^ However, they were predicted to bind two different PpROP4 subunits in the tetramer (Figure 2i). In the complex, PpRopGAP1 exists in a homodimer and the two copies of PpROP4 do not interact. These findings are in line with the notion that purified Arabidopsis AtRopGAP2 dimerizes via its GAP domain and forms oligomers through the CRIB domain.^32^ When PpROP4 was fused with N-mNG, the tetrameric complex could still form, however, the dimerization interface was shifted to include the CRIB domain possibly to avoid a spatial conflict (Figure 2j), which is not consistent with in vitro analysis.^32^ Consequently, the overall complex structure becomes less compact. Thus the oligomerization of RopGAPs and formation of RopGAP-ROP clusters might be impeded. This phenomenon is less likely to occur in the PpROP4-swmNG-PpRopGAP1 complex as swmNG does not contact the interaction site (Figure 2k). Similar to PpRopGAP1, PpRopGEF4 was also predicted to interact with two PpROP4 subunits simultaneously (Figure 2l). However, the structure of the PpROP4-PpRopGEF4 tetramer is not affected by either N-mNG or swmNG (Figure 2m, n). These findings are consistent with the fact that N-terminal tagging reduces but not abolishes membrane enrichment of ROPs. Hence, we concluded that N-terminal tagging may impede the formation of high-order complexes thus reducing stable membrane association of ROPs.

**Figure 3.**
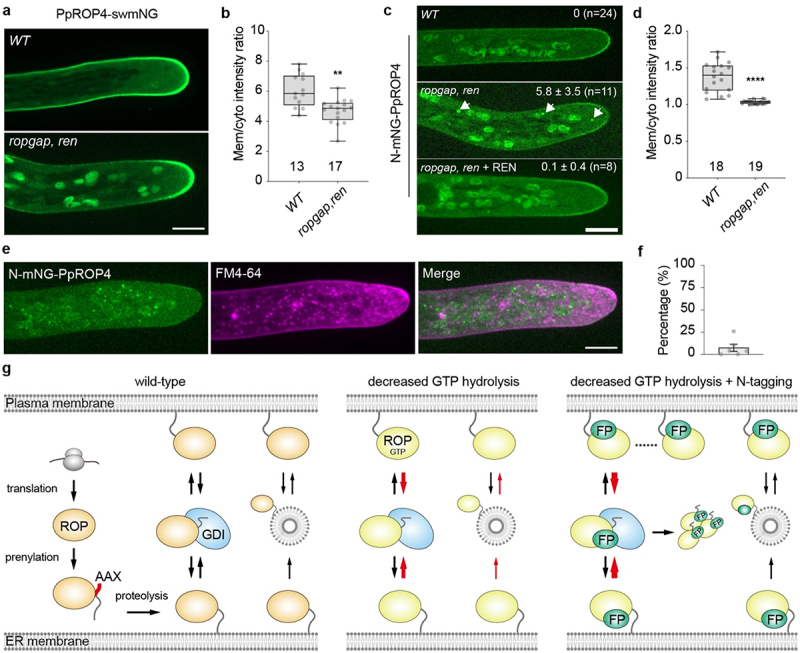
Loss-of-function of ROP-related GAPs impairs membrane localization of N-mNG-PpROP4 and PpROP4-swmNG and a model for the trafficking of ROPs to the plasma membrane. a the localization of PpROP4-swmNG in wild-type (WT) and *ropgap, ren* septuple mutant. b quantification of membrane/cytosol intensity ratio of PpROP4-swmNG. c the localization N-mNG-PpROP4 in WT, *ropgap, ren* mutant and REN overexpressor lines. The average number of observed cytosolic puncta (arrows, mean ± SD) is shown. d quantification of membrane/cytosol intensity ratio of N-mNG-PpROP4. Note that the intensity ratio of N-mNG-PpROP4 is much lower than that of PpROP4-swmNG in WT cells in fig. 3b. Data in b and d are presented as box-and-whisker plots, showing the interquartile range (box), the median (horizontal line), minimum and maximum values (whiskers), and individual data points. The numbers of cells used for quantification are shown at the bottom. Scale bar in a and c: 10 µm. Statistical analyses were performed using two-tailed student’s t-tests. ns, not significant. **, *p* < 0.01. ****, *p* < 0.0001. e FM4–64-labeled vesicles do not colocalize with N-mNG-PpROP4 puncta in the *ropgap, ren* mutant. f percentage of N-mNG-PpROP4 puncta that show colocalization with FM4–64-labeled vesicles. Data are shown as mean ± SD (153 particles from six cells). g a proposed model for ROP GTPase trafficking to the plasma membrane. ROPs are anchored to the ER membrane after translation, prenylation, and proteolysis of the AAX tail in the cytosol. Guanine nucleotide dissociation inhibitors (GDIs) can extract ROPs (likely in both GDP-bound and GTP-bound forms) from the ER to the cytosol and drop off them at the plasma membrane. Both processes are reversible. The membrane-anchored ROPs also travel through the exocytosis pathway to the plasma membrane and can be retrieved through endocytosis. The increase of active ROPs enhances their dissociation from the plasma membrane likely via GDI extraction and has a minor effect on exocytosis (red arrows), leading to a net decrease of membrane-associated ROPs. N-terminal fluorescent tags may impair the clustering of ROPs and their stable association with the plasma membrane, thus increasing the probability of dissociation. This phenotype is exacerbated with the increase of ROP activity and causes protein aggregation in the cytosol (red arrows).

Previously, we have shown that membrane enrichment of PpROP4-swmNG was reduced in the *ropgap, ren* septuple mutant,^24^ suggesting that the activity status of ROPs is critical for polar domain formation. To ask whether the membrane association of PpROP4 also depends on its activity status, we measured the membrane/cytosol intensity ratio of PpROP4-swmNG (see Materials and Methods). As shown in Figure 3a, b, the membrane fraction of PpROP4 is significantly decreased in the *ropgap, ren* septuple mutant. However, this effect is weaker than that caused by N-mNG fusion (Figure 2b). Interestingly, when N-mNG-PpROP4 was introduced in the *ropgap, ren* septuple mutant, its membrane localization was dramatically inhibited (Figure 3c, d). More strikingly, N-mNG-PpROP4 formed puncta-like structures in the cytoplasm, which was never observed with PpROP4-swmNG even in the *ropgap, ren* mutant (Figure 3a, c). These data further support that N-terminal tagging impairs the membrane association of ROP4 and has additive effects when active ROPs are upregulated. Furthermore, when the mutants were complemented with REN overexpression, N-mNG-PpROP4-labeled puncta almost fully reverted to a diffuse pattern (Figure 3c). Hence, these puncta may represent unique subcellular compartments enriched in GTP-bound albeit nonfunctional ROPs.

In yeasts and mammals, the membrane delivery of Cdc42 requires vesicular trafficking^36,37^ and is presumably balanced by endocytosis.^38^ A similar mechanism has also been implicated in plants.^39,40^ Thus we asked whether N-mNG-PpROP4 puncta are vesicles containing trapped PpROP4 due to halted exocytosis and/or enhanced endocytosis. To this end, we stained protonema cells with FM4–64, a lipophilic dye that labels endocytic as well as secretory vesicles derived from recycling endosomes.^41^ Unexpectedly, we did not detect a strong association between N-mNG-PpROP4 puncta and FM4–64-labeled vesicles (Figure 3e, 3f). These data suggest that N-mNG-PpROP4 puncta are caused by defective shuttling of PpROP4 between the plasma membrane and cytosol and this process is sensitive to the global activity status of ROPs.

## Discussion

In this study, we provide evidence that N-terminal tagging impairs ROP function and inhibits its membrane enrichment. Therefore caution should be paid when studying ROP dynamics with an N-terminal fluorescent tag. ROPs have been reported to form nanoclusters within the membrane either under natural conditions or by auxin or osmotic stress treatment.^17–20^ Because N-terminally tagged ROP4 forms protein aggregates only in the *ropgap, ren* mutant but not in wild-type cells, and this phenomenon is not found for internally tagged functional ROP4, whether the clusters formed by N-terminally tagged ROPs are true or induced due to side effects of N-terminal tagging should be carefully evaluated.

Beyond the effects of N-terminal tagging, our data also provide insight into the trafficking mechanisms of ROPs (Figure 3g). In animals, the canonical CAAX-motif-dependent prenylation occurs in the cytosol.^42^ After prenylation, the prenylated targets are attached to the ER membrane where their AAX tails are removed, and they are subsequently delivered to the plasma membrane. The initial processing of ROPs may largely resemble those of Cdc42/Rho/Rac because plant farnesyltransferases that mediate ROP prenylation are present in the cytosol^43,44^ and CAAX processing enzymes that remove the AAX tail and methylate the exposed cysteine residue are all found on the ER membrane.^45,46^

Cdc42/Rho/Rac GTPases could be extracted by GDIs from the ER, relocated into the cytosol, and attached to the plasma membrane.^42^ Alternatively, they are delivered through the exocytosis pathway.^36,37^ Although the GDI extraction model and vesicular trafficking mechanism are non-exclusive, it has been recently proposed that in yeast Cdc42 is primarily delivered to the membrane from the cytosol.^47^ The trafficking of plant ROPs to the plasma membrane is thought to occur through the vesicular trafficking pathway.^4^ This possibility is partly supported by findings that exocytosis-related factors positively regulate the membrane enrichment of ROPs.^39,48–50^ However, ROPs have not been found in vesicle-like compartments under normal conditions, and their vesicular localization has been observed only in a limited number of studies.^39,51^ How much the cytosolic shuttling mechanism and vesicular trafficking pathway contribute to ROP localization remains unclear. The presence of RopGDIs and their ability to regulate ROP localization and membrane association in plants strongly suggest a crucial role of cytosolic shuttling in this process.^20,52–54^ Given that ROPs positively regulate exocytosis,^7,40^ the reduction of membrane-localized ROPs in *ropgap, ren* mutants (Figure 3b) argues against the vesicular trafficking model because a higher activity of ROPs in the mutants should promote exocytosis and enhance their membrane localization. Another possibility is that the increase of active ROPs may enhance membrane extraction by RopGDIs. This is in line with the notion that RopGDIs preferentially bind wild-type and active ROPs in yeast-two-hybrid assays^54,55^ and could efficiently extract them in plant cells.^20^ The extraction of active Rho GTPase is not limited to plant RopGDIs. A recent study has shown that animal RhoGDIs also extract both forms of Rho GTPase,^56^ albeit RhoGDIs are initially found to bind inactive Rho GTPases in human.^57^

Why do N-terminally tagged ROPs form cytosolic puncta in *ropgap, ren* mutants but not in wild-type cells? N-terminal tagging appears not to strongly influence the activity status and GTP-bound conformation of ROPs because ROP fusion proteins have high GTPase activity and binding capacity with regulatory proteins in vitro^24,34^ and do not exhibit a significant change in predicted heterodimeric structures with activity regulators (Figure 2e-h). However, N-terminal tags have a great potential to impede the formation of high-order complexes (Figure 2i-k), which may inhibit cluster formation and destabilize the membrane association of ROPs. Presumably, the unclustered ROPs are more prone to extraction by RopGDIs. Therefore, we propose that N-mNG-PpROP4 puncta are induced by enhanced RopGDI extraction due to the increase of active ROPs and their inability to form stable protein clusters on the membrane. Although the involvement of vesicular trafficking could not be completely excluded, the absence of colocalization between N-mNG-PpROP4 puncta and vesicles disfavors this possibility (Figure 3e, f). In summary, our findings suggest that cytosolic shuttling may play an important role in regulating membrane localization of plant ROPs and this process requires tight regulation of ROP activity status. This model has been also proposed for Cdc42 in yeasts^47^ and might be conserved in eukaryotes.

## Data Availability

All data generated or analyzed during this study are included in this published article.
